# Acute effects of a multinutrient supplement on performance, rate of perceived exertion and markers of catabolism in young resistance trainers practitioners

**DOI:** 10.1186/1550-2783-11-S1-P9

**Published:** 2014-12-01

**Authors:** Marcos Seijo, Nadia Ashrafi, Joshua Smith, Christian Wilkinson, Yue Fu, Jack Miller, Eneko Larumbe, Fernando Naclerio

**Affiliations:** 1 Centre for Sport Science and Human Performance, School of Science, University of Greenwich, London, United Kingdom; 2 Department of Pharmaceutical, Chemical and Environmental Sciences, School of Science, University of Greenwich, London, United Kingdom; 3 Fundamentals of Motricity and Sports Training Department, School of Sports Sciences, European University of Madrid, Madrid, Spain

## Background

The ingestion of a multi-nutrient containing proteins, carbohydrate and creatine has been shown to enhance acute and chronic responses to resistance training, attenuating fatigue and markers of catabolism training after resistance training workouts. The purpose of this study was to investigate the acute effects of a commercially available multinutrient supplement on neuromuscular fatigue, performance, perception of effort and salivary markers of catabolism, during a bout of resistance circuit training session.

## Methods

Twelve recreationally resistance trained young males (age 22±1.5 years, body weight 79.4±10.2 kg, height 181±0.07 cm), volunteered to participate in the study, completing 2 randomised controlled circuit resistance training sessions (CT). Immediately before and after the workout, participants consumed 500ml of water mixed with 60g of a multinutrient supplement (MTN) containing whey proteins, carbohydrate, creatine, HMB and sodium bicarbonate, or maltodextrin (PL). CT involved three rounds of 7 resistance exercises (CMJs, Bench Press, Parallel-Squat, Upright row, Alternate Lunges, Dead Lift, Push-press, Abdominals) followed by 1 min rest. Participants performed 12 repetitions at 70% 1RM in each of the exercises with no rest in between (only the time to change from one exercise to the next). Measurements included total kg lifted per exercise and in the overall workout, the rate of perceived exertion (RPE) determined at the end of each circuit, pre and post blood lactate and markers of neuromuscular fatigue, including Countermovement Jump (CMJ), 1RM Bench Press (1RMBP) and the maximal velocity at 50% of 1RM Bench Press (V50%BP). In addition, salivary markers of catabolism: Free Testosterone (T) and Cortisol (C) were assessed pre, 30min and 60min post CT. Consent to publish the results was obtained from all participants.

## Results

No significant differences were observed between the total weight (kg) lifted for the entire CT or exercise (P>0.05). RPE increased significantly during CT (p<0.05), but without differences between conditions. Lactate increased significantly from pre to post in both conditions (p<0.05), but without differences between them. Markers of neuromuscular fatigue (CMJ; 1RMBP and V50%BP) significantly decreased from pre to post but without difference between conditions. Salivary C showed a trend, increasing from pre (7.9±6.2 nmol/L) to 30min post (18.8±12.7 nmol/L -p=0.60-) and 60min post (18.1±14.7 nmol/L -p=0.93-) in PL while no significant changes or trends were observed for MTN (10.1±7.0 Vs 16.6±13.9 and 12.8±8.5 respectively). T showed significant higher values at 30min post (510.1±124.9 pmol/L -p=0.005-) compared to pre (360.4±104.6 pmol/L) and a returned to baseline levels at 60min post (402,1±88,4 pmol/L) for PL, while no changes were observed for MTN. In addition, cortisol showed a strong tendency to be higher for PL compared to MTN at 60min post (18.1±14.7 Vs 12.8±8.5 nmol/L -p=0.054-). No other differences or trends were observed.

## Conclusion

Ingesting a MTN supplement immediately before and after a circuit resistance training workout, resulted in no impact on performance, attenuate neuromuscular fatigue or perception of effort with respect to the ingestion of only carbohydrate. However, some effect to attenuate the rise in cortisol (30 min and 60 min post) and testosterone (30 min post) could be produced.

